# Echolocation calls and communication calls are controlled differentially in the brainstem of the bat *Phyllostomus discolor*

**DOI:** 10.1186/1741-7007-3-17

**Published:** 2005-08-01

**Authors:** Thomas Fenzl, Gerd Schuller

**Affiliations:** 1 Max Planck Institute of Psychiatry, Kraepelinstr. 2-10, Munich, 80804, Germany; 2 Department Biology II, Ludwig-Maximilians-Universitaet, Grosshaderner Str. 2, Planegg-Martinsried, 82152, Germany

## Abstract

**Background:**

Echolocating bats emit vocalizations that can be classified either as echolocation calls or communication calls. Neural control of both types of calls must govern the same pool of motoneurons responsible for vocalizations. Electrical microstimulation in the periaqueductal gray matter (PAG) elicits both communication and echolocation calls, whereas stimulation of the paralemniscal area (PLA) induces only echolocation calls. In both the PAG and the PLA, the current thresholds for triggering natural vocalizations do not habituate to stimuli and remain low even for long stimulation periods, indicating that these structures have relative direct access to the final common pathway for vocalization. This study intended to clarify whether echolocation calls and communication calls are controlled differentially below the level of the PAG via separate vocal pathways before converging on the motoneurons used in vocalization.

**Results:**

Both structures were probed simultaneously in a single experimental approach. Two stimulation electrodes were chronically implanted within the PAG in order to elicit either echolocation or communication calls. Blockade of the ipsilateral PLA site with iontophoretically application of the glutamate antagonist kynurenic acid did not impede either echolocation or communication calls elicited from the PAG. However, blockade of the contralateral PLA suppresses PAG-elicited echolocation calls but not communication calls. In both cases the blockade was reversible.

**Conclusion:**

The neural control of echolocation and communication calls seems to be differentially organized below the level of the PAG. The PLA is an essential functional unit for echolocation call control before the descending pathways share again the final common pathway for vocalization.

## Background

Bats use echolocation calls for orientation in space and hunting for prey. Communication calls serve the purpose of inter- and intraspecific social communication. Neural control of both types of calls has access at the level of the medulla to the final common pathway for vocalization. Motoneurons controlling the larynx, the vocal tract and the respiratory muscles are accessed via interneurons from the nucleus ambiguus/retroambiguus complex [1]. One major subcortical structure for the control of vocalizations above the level of the medulla is the periaqueductal gray matter (PAG). Here, communication calls can be elicited by electrical or pharmacological stimulation in many mammalian species like squirrel monkey [2], rhesus monkey [3], rat [4], guinea pig [5], gibbon [6] and bat [7,8]. While in most areas where vocalizations can be elicited, long persisting stimulation causes a fading of the vocal reaction, the PAG is one of the very few regions where natural communication calls can be elicited without changes in the emotional state of the animal and without habituation to the stimulus, even over extended stimulation periods [9]. PAG stimulation in bats also evokes echolocation calls [10,11] without motor concomitants (except ear and nose leaf movements), and without habituation to the stimulus [7]. The PAG in bats is thus involved in the control of communication calls as well as echolocation calls. The different types of calls are elicited at anatomically distinct PAG locations [7].

In monkeys, the PAG has been shown to be part of the vocalization controlling system [12]. Although the author found the majority of vocal- eliciting structures within the limbic system, only stimulation in the PAG yielded all types of communication calls used by the animal [12].

Further findings suggest a hierarchical organization of the vocalization controlling system including the PAG. Bilateral lesions of vocalization-eliciting areas in the forebrain and diencephalon do not affect vocalizations triggered in the PAG, whereas lesions made at brain levels below the PAG, e.g., in the dorsolateral pons and the ventrolateral medulla suppress PAG-triggered vocalizations [13]. The PAG would thus serve a gating function controlling the release of vocal patterns that themselves are constituted in networks below the PAG level [14].

In the bat (*Rhinolophus rouxi*), three areas below the midbrain level in addition to the PAG have been identified that yield echolocation calls (but not communication calls) on electrical stimulation without temporal or spectral distortions [18]: a) the deep layers of the superior colliculus, b) the deep mesencephalic nucleus in the reticular formation and c) an area medial to the rostral parts of the dorsal nucleus of the lateral lemniscus, the paralemniscal area (PLA). All three areas share most of the stimulus-response conditions found in the PAG, such as very short latencies of vocalizations on stimulation, no habituation to stimulus, no body movements accompanying the vocalizations and no distortion of the call pattern. However, only echolocation (and not communication) calls can be elicited from these areas. Moreover, echolocation calls from the PLA can also be elicited by microinjection of a glutamate agonist (kainic acid), indicating that it is the neurons in the PLA rather than fibers passing through this area that are responsible for the activation of vocal utterances [19].

The PLA displays similar properties in other bat species, e.g., *Pteronotus p. parnellii *[20] and *Phyllostomus discolor *[7,21]. The interest in the PLA as an important component for vocal control in echolocation was supported by the finding that neurons in the rostral portion of the PLA of *R. rouxi *were active both before and during vocal emissions and also showed simultaneous responses to auditory stimuli. The PLA could therefore serve as an audio-vocal interface between auditory processing and motor control of vocalization [22,23]. The physiological data on the PLA available from animals other then bats are very sparse and anatomically corresponding areas have been investigated under different functional aspects [23-28].

Vocal control in other, non-echolocating mammals is characterized by a rather direct connection from PAG to the ambiguus/retroambiguus complex [1,15-17] or to the reticular formation [13,14,29], which would not necessitate an interaction from other brain structures (e.g., PLA) below the PAG level. But these findings seem to be insufficient to explain findings from Schuller and coworkers and Fenzl on the involvement of the PLA in motor control of vocalizations. A parallel organization of the descending control system for vocalizations could be assumed at the level of the PAG and PLA, and it is of interest whether this organization is a general mammalian feature.

The present study investigates whether the PAG and PLA interact during the PAG-induced emission of communication and/or echolocation calls in *P. discolor *using reversible blockade of PLA function. We found that PAG-induced communication calls could not be blocked either through ipsilateral or contralateral blockade of the PLA. However, PAG-induced echolocation calls could be temporarily blocked only through contralateral blockade of the PLA. These differences display partly differentiated functional organizations of vocal controlling pathways for communication calls and echolocation calls below PAG level.

## Results

### Implants – Stability of chronical microstimulation

**Figure 1:**
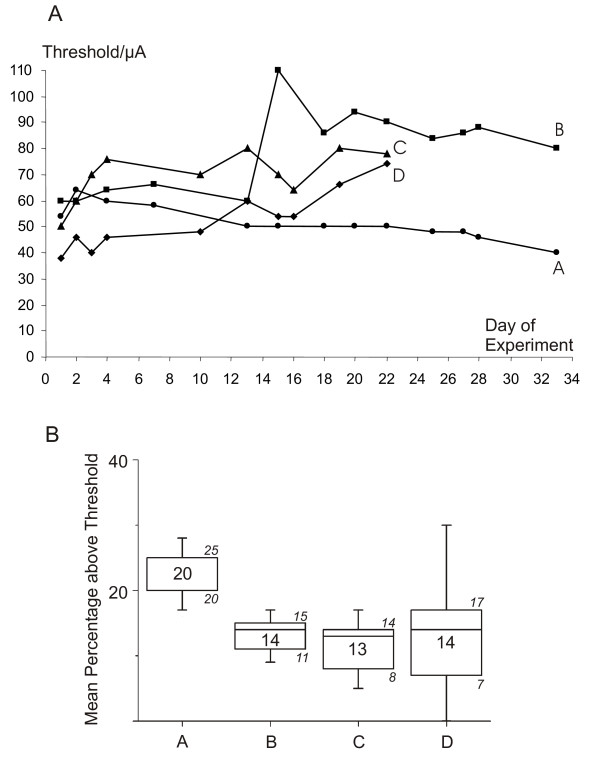
**Stability of implants. **Stability of stimulation threshold currents to elicit vocalizations and time course at chronic microstimulation probes. Electrodes were implanted at day0 of the graph A. (A) Thresholds of four chronically implanted stimulation probes (A/B and C/D) are shown. Stimulation through implant A and B (animal 1) and through implant C (animal 2) elicited echolocation calls, while stimulation through implant D (animal 2) triggered communication calls. (B) "Mean percentage above threshold"-values for all four implants (A to D) as plotted in A during the actual blockade experiments. The mean stimulation current was 20% (electrode A), 14% (electrode B), 13% (electrode C) and 14% (electrode D) above threshold to ensure a vocal answer on each stimulus within the pulse train. At threshold level, not all stimuli triggered a vocalization. The median values are indicated as numbers; additionally P25 and P75 values are shown in italics.

Echolocation calls and communication calls were reliably elicited by electrical stimulation at specific stimulation sites in the PAG of *P. discolor *using chronically implanted stimulation electrodes. The thresholds for eliciting vocalizations varied within relatively narrow limits from probe to probe and from one experimental day to the next. In Fig. 1A the temporal variation of thresholds are plotted for the four implants over the entire experimental duration of 22 (animal 1) and 33 days (animal 2), respectively. The thresholds remained relatively stable between the day 4 and day 13, whereas stronger changes occurred in the first days after implantation or in some cases later than 13 days. Overall deviation between the day of implantation and the last experimental day was -30%, +33%, +56% and +95% in experiments A, B, C and D, respectively. During experiments the stimulation current was adjusted at supra-threshold levels to provide a reliable one-to-one relationship between electrical stimuli and vocal responses. The median values of supra-threshold currents used with the chronical implants A, B, C and D were 20% (P25 = 20, P75 = 25), 14% (P25 = 11, P75 = 15), 13% (P25 = 8, P75 = 14) and 14% (P25 = 7, P75 = 17) above threshold, respectively (Fig. 1B).

### Echolocation calls versus communication calls

In order to test whether the production of echolocation or communication calls could be suppressed by neuropharmacological blockade, the nonselective glutamate antagonist kynurenic acid (KA) was iontophoretically injected into the PLA. Injections were either ipsilateral or contralateral to the PAG stimulation site. Twelve blockade experiments were conducted with consistent results in animal one (implants A and B), and seven experiments were conducted in animal two (implants C and D).

**Figure 2:**
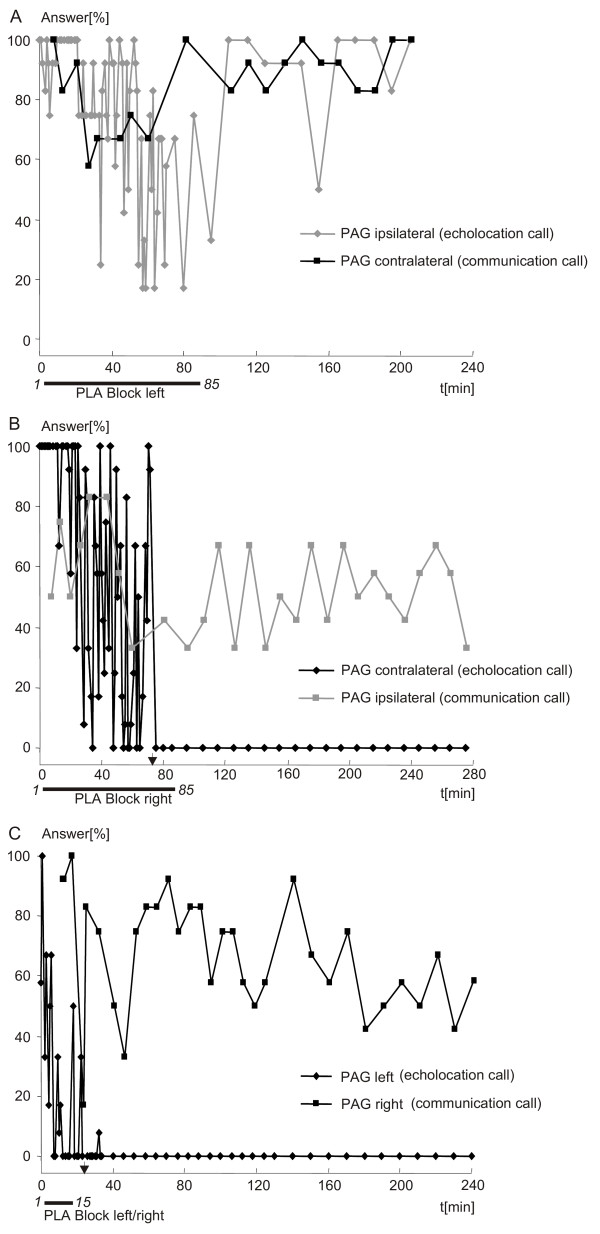
**PLA-located blockade of PAG-induced echolocation calls and communication calls. **The percentage of successful stimulations (y-axis) for eliciting vocalizations in the PAG is represented during and after KA induced blockade of PLA. Echolocation calls are graphed with diamonds, while squares are used for communication calls. The kynurenic acid application is indicated by black horizontal bars below the x-axis. Onset and termination times of iontophoresis are given in italics. Onset and termination times of iontophoresis are indicated by numbers at both ends of the bars in italics. Stimulation success (%) was calculated for intervals of 2 s. Black arrows on the abscissa indicate the 25% blockade boundary. (A) Application of KA to the left PLA. The ipsilateral production of PAG-induced echolocation calls (left electrode) cannot be blocked, although a slight depression can be noticed in the first half of the graph. The contralateral production of PAG-induced communication calls (right electrode) is only rarely influenced by the glutamate antagonist KA. (B) A blockade of the right PLA totally blocks the production of contralaterally PAG-induced echolocation calls (left electrode), while the ipsilateral production of communication calls (right electrode) again is barely influenced. Note that the curve for PAG ipsilateral (communication call) starts around 50%. (C) Bilateral blockade of both PLA sites again leads to a total depression of PAG-induced echolocation calls, while PAG-induced communication calls can be elicited across the entire experimental run. Prior to each experiment, vocal answers were stable for at least 10 minutes at a value comparable to the data shown at the start of the application of the antagonist.

Blocking the PLA with KA suppressed PAG-induced echolocation calls whereas PAG-induced communication calls were less affected or not affected at all. The degree of suppression of echolocation calls depended on whether the PLA blockade was applied ipsilaterally or contralaterally to the PAG stimulation sites as shown in Fig. 2. An 85 min application of KA to the left PLA, i.e., the PLA ipsilateral to the PAG electrode eliciting echolocation calls and contralateral to the electrode triggering communication calls, did not completely suppress either echolocation calls (Fig. 2A, gray) or communication calls (Fig. 2A, black). A reduction of response probability for echolocation calls could, however, be seen around 50 min after the onset of iontophoresis. The activation probability for communication calls varied largely around 50%, and showed only a relative persistent lowering to around 40% between 50 and 90 min after the onset of KA application (Fig. 2B, gray).

A much different pattern of vocal responses appeared if the block was applied to the PLA contralateral to the PAG electrode eliciting echolocation calls. The response probability for echolocation calls started to decrease about 10 min after the onset of KA application and showed great variability, until complete suppression at around minute 80 (Fig. 2B, black). The blockage persisted beyond the termination of the experiment after 280 min.

When KA was applied bilaterally to both PLA sites, the suppression of PAG-induced echolocation calls occurred with a much shorter delay (about 24 min). However, PAG-induced communication calls could not be completely suppressed, although the probability of eliciting communication calls showed generally greater variability in response to KA application (Fig. 2C). A slight effect of KA could be discerned around 30 min after onset of the application; however, the effect did not persist. The slight mean decrease in elicitability, i.e., not falling below about 50%, and the high variability of elicitation probability persisted throughout the entire duration of the experimental run. These effects lasted beyond the 240 min, after which the experimental sessions had to be terminated.

After each blocking experiment a recovery period was inserted. Subsequently, a control experiment with electrical microstimulation only was started 24 hrs after the beginning of the previous experiment. In all control runs the elicitability of both types of calls had fully recovered. Calls could be elicited at standard stimulation currents, and no lesioning of PLA structures by iontophoretic currents could be detected. Typically the next iontophoresis experiment started within 24 hrs after the control experiment.

### Ipsilateral vs. contralateral blockade of echolocation calls

**Figure 3:**
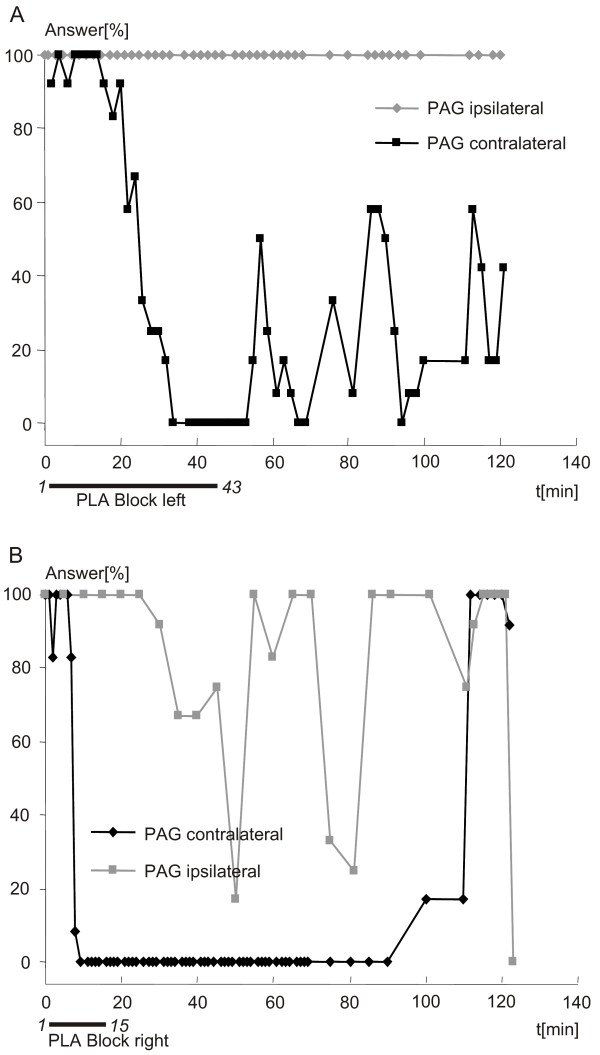
**PLA-located blockade of PAG-induced echolocation calls. **At both electrodes, echolocation calls were triggered. Refer to Fig. 2 for explanations on graph. (A) Application of kynurenic acid to the left PLA does not influence the elicitability of ipsilaterally PAG-induced echolocation calls (left electrode), whereas it lowers dramatically the efficiency of contralaterally induced echolocation calls (right electrode). The elicitability does not recover to the 75% mark within termination of the experiment at minute 120. (B) A blockade of the right PLA totally blocks the production of contralaterally PAG-induced echolocation calls (left electrode), while ipsilateral-induced echolocation calls (right electrode) are little affected (although some drop in elicitability can be detected). Prior to each experiment, vocal answers had to be stable for at least 10 min at a value comparable to the data shown at the application of the antagonist.

The finding that a PLA-block by KA primarily affects contralaterally PAG-induced echolocation calls was supported by the results from following experiments, shown in Fig. 3. First, KA was applied to the left PLA for 43 min. Echolocation calls elicited in the PAG contralateral to the iontophoresis site were completely depressed about 32 min after the onset of iontophoresis and partially recovered about 10 min after termination of KA application. In contrast, the ipsilateral PAG-triggered echolocation calls were not affected at all. Similarly, the right PLA was blocked by KA application for 15 min. Again, the contralateral PAG-induced echolocation calls were blocked consistently about 7 min after iontophoresis onset and started to recover about 80 min after cessation of KA application. Echolocation calls elicited in the ipsilateral PAG were temporarily impeded, but elicitability was far from being totally blocked.

Although the depression of PAG-induced echolocation calls persisted beyond the end of the experimental session (140 min; Fig. 3A), elicitability from both PAG electrodes was fully recovered in the control experiments 24 hrs later.

### Influence of sedation on animal during experiments

To ensure a stable position of the animal during the iontophoresis experiments, Rompun^® ^was chronically applied subcutaneously during the sessions. Sedative was adequate to achieve optimal stability of the animal. Both echolocation calls and communication calls were elicited consistently over a long stimulation period of up to 33 days without any change in spectral composition of the calls. Motor reactions associated with vocalizations, such as pinna and nose leaf movement with echolocation calls or mouth movements with communication calls, also did not change during the experiments.

**Figure 4:**
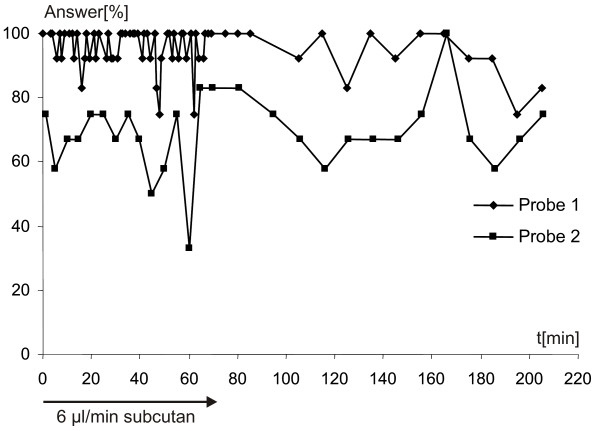
**Sedation has no influence on electrical stimulation. **Influence of sedation on vocal performances. Prior to electrical stimulation, an initial dose of 0.5 ml sedative (0.04% Rompun^® ^in 0.9% NaCl) was injected subcutaneously. Starting 30 min later at minute 0 of the plot, a continuous dose of 6 μl/min of 0.04% Rompun^® ^was infused subcutaneously for a total of 205 min. The elicitability of electrically triggered PAG vocalizations is shown for a time period of 205 min. Compared to electrically triggered vocalizations before the sedative was injected (data not shown), no difference can be detected. Therefore, the sedative has no influence on electrically elicited vocalizations. Probe 1 and 2 refers to two implanted electrodes.

To ensure that sedation had no influence on activation of electrically induced vocalizations, a pure stimulation experiment under identical depressant conditions as applied at the blockade experiments was carried out (Fig. 4). Neither the initial dose of 0.5 ml sedative (0.04% Rompun^® ^in 0.9% NaCl) prior to the experiment nor the continuous application of 6 μl/min of sedative showed any influence on the ability to trigger vocalizations. The vocalizations triggered under the influence of the sedative in general showed no difference to vocalizations triggered without sedation or to vocalizations emitted spontaneously, either in spectral or temporal patterns. Also the percentage of vocal answers triggered by one pulse train (duration: 2s, 12 single stimuli, 12 vocal answers correspond to 100% vocal answer) did not decrease under the influence of the sedative, as compared to experiments without sedation (data not shown). A decline or even depression of vocal answers of the type seen in the blockade experiments could not be detected.

### Histological verification of electrode positions

**Figure 5:**
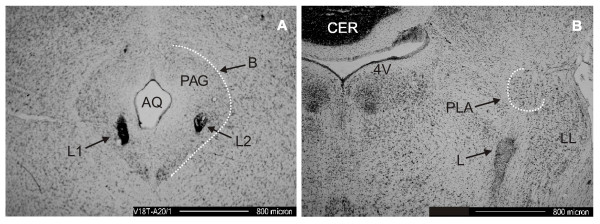
**Electrical lesions. **Histological verification of electrode locations A/B show Nissl stained 42 μm frontal sections. (A) Two lesions (L1/L2) caused by repeated electrical stimulation through chronically implanted electrodes placed into vocally active sites within the PAG. AQ, aqueduct; B, boundary between PAG and surrounding tissue. (B) Electrically induced lesion (L) 400 μm below the location of the iontophoresis probes in the PLA. Due to the 400 μm offset of the lesion below the PLA the function of the PLA during further experiments was not influenced. CER, cerebellum; LL, lateral lemniscus; 4V, 4^th ^ventricle.

Stimulation sites in the PAG and in the PLA were identified by tissue lesions (Fig. 5). The lesions in both structures could easily be detected in anatomical sections.

## Discussion

This study has demonstrated that the neural control of echolocation calls and communication calls must have access to at least partly different neural substrates for vocalization.

### Sedation of animals, stability of chronic implants and iontophoretic efficiency

The stability of the chronically implanted electrodes for electrical micro-stimulation was very satisfactory. Both echolocation calls and communication calls were elicited consistently over a long stimulation period of up to 33 days without any change in spectral composition of the calls. The slight increase of stimulation thresholds for the individual electrodes (Fig. 1) could be attributed to an accumulation of glia cells and debris caused by the presence of implants. Motor reactions associated with vocalizations also did not change during the experiments.

It is noteworthy that the onset of a contralateral PLA blockade is extraordinarily variable between 3 to almost 80 min under comparable experimental conditions (Fig. 2 and Fig. 3). Slightly different positions of the iontophoresis probe at the PLA site may be responsible for this, as even deviations as small as 100 μm correspond to almost 15% of the mediolateral dimension (≈ 800 μm, [7]) of the PLA in *P discolor*. At marginal application sites, KA would have taken longer to influence the necessary number of neurons in the PLA than when injected the geometrically optimal PLA location. The onset time differences indicate that the suppressive effect of PLA inactivation depends on the of PLA neurons.

### Differentially organized vocal substrates for the production of echolocation calls and communication calls

Vocalizations are complex motor patterns imbedded into differentiated behaviors of an animal. It is well established that the PAG plays an important role in vocal control of communication calls [14,30], e.g., communication calls can be triggered by stimulating the PAG in several mammalian [7,31,32] and non-mammalian species [33]. In addition, echolocation calls can be elicited in bats within restricted areas of the PAG [8] that are distinct from areas in which communication calls can be triggered [7].

Outside the PAG, echolocation calls can also be elicited in a variety of brainstem areas [18], among which the PLA shows the lowest thresholds and the shortest latencies for eliciting ultrasonic vocalization. However, no communication calls can be elicited from the PLA [7,18,20]. From these findings, the hypothesis was deducted that different types of vocalizations could be modulated via at least partially separate and/or parallel vocal pathways in the bat.

A vocal pathway from the PAG to the nucleus retroambiguus (NRA) for the production of communication calls has been neuroanatomically defined by several authors [1,16,32,34]. The NRA includes a group of premotor neurons which send direct projections to thoracic and upper lumbar motoneurons [17] involved in expiration, and to the nucleus ambiguus containing laryngeal and pharyngeal motoneurons [15,35]. According to current knowledge, the PLA has no direct interferences with components of this pathway. The direct descending vocal path therefore cannot account for the blockade of PAG-induced echolocation calls by inactivation of the PLA as demonstrated in this paper.

Inactivation of confined areas in the brainstem exerts suppressive action on vocalization also in other mammals besides bats, as demonstrated by Jürgens [36]. Here, KA injections into the ventral pons of squirrel monkeys blocked a specific type of PAG-triggered communication call with characteristic frequency modulations, whereas other call types remained unaffected.

Jürgens suggests that vocal patterns are generally controlled in different brainstem regions, and that vocalizations with frequency modulations seem to depend on an intact periolivary region [36]. Echolocation calls of *P. discolor *are typically frequency-modulated calls covering a range between 45 and 100 kHz with the 3^rd ^– 5^th ^harmonic [37]. Our findings support the assertion by Jürgens that different vocal patterns could be controlled or modulated through activity in different brainstem regions, at least pertaining to echolocation calls and particular types of communication calls.

The effect of PLA-induced blockades on PAG-triggered vocalizations strictly depends on the side of the application, since echolocation calls can only be blocked when KA is applied contralateral to the stimulation site in the PAG. This is in contrast to what Jürgens describes for squirrel monkeys [36] where PAG-elicited vocalizations were only affected by ipsilateral, and not contralateral KA injection into the ventrolateral pons.

This difference in functional laterality may be attributed to the different brain regions involved in both studies and their specific connectivity. Since the neuroanatomical connectivity of the PLA is far from being understood, there is no straightforward explanation of this heterolateral influence. Besides strong reciprocal connections between the PLA [18,20] of both anatomical sides, there are no major midline crossing projections known so far to our knowledge that could account for the contralateral influence. Until today no anatomical data are available to describe connections between the PAG and the PLA and from the PLA towards the region of the nucleus retroambiguus/ambiguous complex in *P. discolor*. Only very few data are available from other species. In *P. p. parnellii*, efferents to the PAG were found when wheat germ agglutinin conjugated to horseradish peroxidase (WGA-HRP) was injected into vocally active sites of the PLA [20]. Efferents to the nucleus ambiguus from the PLA were found by Metzner using WGA-HRP [38] and connections from the lateral tegmental area (LTR) to the PAG and contralateral LTR were found using WGA-HRP and fluorescent tracers [27,28]. However, these data are not sufficient to explain the effectiveness of contralateral blockades under anatomical aspects.

The enhancement of the suppression effect by bilateral KA application may have two reasons. First, reciprocal interconnection between the two PLAs has been shown anatomically [20]. Second, ipsilateral blockage of the PLA also leads to reduced probability for eliciting echolocation calls, although it never reaches the level of suppression seen in most contralateral cases. The reciprocal interaction between the two PLAs is predominantly inhibitory as shown in *R. rouxi *(Schuller, unpublished), but the functional significance of this interaction is unclear. The assumption of a bilateral, but strongly unbalanced, descending control of echolocation calls via the PLA seems to fit the data more closely than does the assumption of a strictly unilateral organization. The presence of a non-functional ipsilateral PLA in addition to the silent contralateral PLA would further and more effectively decrease the probability for eliciting echolocation calls, resulting in a shorter time for onset of supression.

Based on connectional evidence (i.e., direct projections from the lateral part of the caudal PAG to the nucleus retroambiguus in the cat), Holstege proposed that the vocal pattern generation takes place within a final common pathway for vocalization driven by input from the PAG [15-17]. However, these findings and those of Zhang [1] that the vocal control pathway consists only of a direct connection from the PAG to the nucleus retroambiguus in the medulla oblongata are not supported by the results from the monkey [36]. Likewise, while our findings do not rule out a direct connection from the PAG to the nucleus retroambiguus (certain types of communication calls), they also demonstrate that a differentiated control for vocalizations (echolocation calls, frequency modulated) via parallel or at least partly separated pathways for echolocation calls could exist. This evidence of a more complicated network for vocal control at the level below the PAG in the monkey, as well as in the bat, underlines the broader significance of this concept on a mammalian level. The bat vocal control system therefore cannot be considered to be specialized, but is a general mammalian vocalization system with distinct emphasized features.

## Conclusion

Communication calls and echolocation calls can be elicited with electrical microstimulation through chronically implanted electrodes at different sites within the PAG. Reversible blockade of the vocally active PLA in the region in which only echolocation calls can be triggered totally blocks PAG-induced echolocation calls but not communication calls. Thus, the PAG-NA/NRA pathway for vocalization described in literature may not be the only pathway processing vocal activity. The PLA seems to be essential for the production of echolocation calls but not for particular types of communication calls elicited in the PAG. This suggests differential pathway organization for particular types of communication calls on one hand and echolocation calls on the other hand. Whether the differentiation of pathways applies to the two classes of echolocation calls and communication calls in general, or whether it is more directly dependent on specific call properties in the acoustic pattern domain, remains open to further experimentation.

## Methods

### Experimental design

Two male neotropical bats (*P. discolor*) originating from the departmental breeding colony were used for this study. During the experiments, the animals were kept under semi-natural conditions with a 12:12 hrs light cycle, at 70% relative humidity and 28°C.

Surgical preparation of the animals was done under 4% Isoflurane (CuraMED Pharma, Karlsruhe, Germany) anesthesia. After additional local application of 2% Xylocain^® ^(Astia, Germany), skin and muscles overlying the skull were cut along the midline, retracted to the sides and stabilized with sponge material (Gelastypt^®^, Hoechst, Germany). Minor bleedings were stanched with the coagulant Orbat^® ^(lege artis Pharma, Germany). The skull surface was freed from tissue remains and a small holding tube was attached with a light-curing dental compound (Microglass^®^, Kulzer, Germany).

Experiments were conducted in an anechoic chamber, thus reducing acoustical interferences from the environment and reflections of call signals. The animals were placed in a holder that prevented gross body movements and the head was immobilized by attaching the surgically affixed tube to a head holder that allowed accurate repositioning (≤ 10 μm) of the animal in the stereotaxic device throughout repeated experimental procedures.

The orientation of the skull and consequently the brain within the stereotaxic coordinates was determined by scanning the profile lines of the exposed skull in parasagittal and transverse directions at the first post-operative day. Details of the stereotaxic device, the procedures to determine the skull position, and the reconstruction of the stimulation and iontophoresis sites are described elsewhere [39]. This method typically yields accuracy better than150 μm in all three dimensions.

Localization of vocally active sites within the PAG and PLA by electrical microstimulation typically started on the third postoperative day. Thirty minutes before each experimental session, an initial dose of 0.4 ml sedative (0.04% Rompun^®^/0.9% NaCl) was injected subcutaneously. The sedated state was maintained by continuous subcutaneous infusion of 0.04% Rompun in physiological saline with a rate between 3.5 μl to 5.5 μl/min (syringe pump: TSE-Systems, Bad Homburg, Germany). Sessions were generally limited to a maximum of 5 hrs per day.

Stimulation electrodes and iontophoresis probes were inserted through small holes of a typical diameter of 200 μm. Penetrations were made at different rostrocaudal and mediolateral inclinations in order to reach different locations through the same hole. All coordinates of probe positions were referred to the reference point of the equipment also used to determine skull and therefore brain position, and thus could be mathematically transformed to coordinates of standard frontal sections, corresponding to a specially prepared standard working brain atlas for this bat species (T. Fenzl and A. Nixdorf, unpublished). For further verification, stimulation and iontophoresis sites within the brain were marked using electrical lesions (-4 μA DC for 5 min) through the electrodes implanted in the PAG and through the stimulation electrodes used to localize the vocally active sites in the PLA.

### Electrical microstimulation

For acute microstimulation, Parylene-coated tungsten electrodes (type TM33A20, WPI Inc., Sarasota, USA) were used. Teflon-insulated silver wires (AGT0510, WPI, Sarasota, USA, diameter: 125 μm) were implanted for chronic PAG micro-stimulation. A sharpened tungsten wire inserted into the neck musculature served as an indifferent electrode.

Electrical stimuli consisted of 15 ms long bursts with 0.1 ms long negative rectangular current pulses at 1 kHz rate, and were presented at a repetition rate of 6 bursts per s (stimulatorS48 with stimulus isolation unit PSIU6, Grass, Quincy, USA). One stimuli train lasted 2s.

During experiments, the animals were continuously monitored by TV under infrared light illumination (camera: Teli, Tokyo, Japan; monitor: TC-800 E4D, Osaka, Japan)) (LED array: 12 V/28 LED; Conrad Electronics, Germany).

### Chronic stimulation electrodes

Sharpened Teflon^®^-insulated silver wires(type AGT0510, WPI, Sarasota, USA) with a wire diameter of 125 μm and 100–200 μm bare length were secured at the skull with light curing dental compound cement at PAG positions at which appropriate calls (communication and echolocation calls) could be electrically elicited. A pair of IC-socket pins was used as connectors. The electrical stimulus could be switched between electrodes using a remote controlled electric relay, interposed between the isolation unit and the stimulation electrodes.

A graphical overview of the experimental approach is provided in the additional file (Additional file 1).

### Iontophoresis within PLA

For reversible blockade of the PLA, the glutamate antagonist kynurenic acid (KA; 75 mM at pH 9; Sigma-Aldrich, Steinheim, Germany) was iontophoretically applied through borosilicate glass microelectrodes with a tip diameter between 3 μm and 5 μm. Retaining current was 30 nA and ejection currents ranged between 200 nA and 250 nA, delivered by a Neurophore BH-2 system (Medical Systems Corp., Greenvale, USA).

PAG-triggered vocalizations were considered to have been blocked by iontophoresis in the PLA when 75% of electrical stimulations failed to elicit vocalizations during a stimulation period of 10 min (coherent).

### Sound recording, processing and data analysis

Vocalizations were picked up with an ultrasonic microphone (type 4135, Bruel & Kjaer, Darmstadt, Germany), amplified, digitally converted at 250 kHz sample rate (CIO-DAS16/M1, Computer Boards Inc., Mansfield, USA), and stored on a personal computer. The recording program was written in Agilent-VEE (version 6 pro, Agilent, USA). Spectral analysis of the recorded vocalizations for call identification and evaluation of frequency shifts was performed with the software "Bat Sound" (Petterson Electronic AB, Sweden). Peak amplitude of vocalizations was derived from the power spectrum of vocalizations. Call length was evaluated using the envelopes of the vocalizations.

### Neuroanatomical processing

The animals were euthanized at termination of the experiments with Nembutal^® ^(16 mg/100 g BW) and transcardially perfused with 4% paraformaldehyd in 0.05 M phosphate buffer solution (PBS). The brains were soaked in 30% sucrose in 0.05 MPBS for cryoprotection. For embedding with egg yolk, the brains were aligned in a small Perspex chamber so that the section plane would best correspond to that of the reference brain sections of the brain atlas [39]. The brains were cut on a cryostat (Frigocut type 2700, Reichert-Jung, Germany) into 42 μm slices and generally three adjacent series were processed. Routinely, Nissl and fiber stains [40] were applied for identification of stimulation sites. The standardized cutting procedure made it possible to refer anatomical data from individual brains to the sections of the reference brain, permitting a comparison of data from individual animals.

### Animal care

Principles of laboratory animal care were followed and experiments were conducted under the regulations of the current version of German Law and Animal Protection. Reference Government of Bavaria (Az.Reg.v.Obb.211-2531-37/98).

## Authors' contributions

TF developed chronic implants and designed and carried out all experiments. GS participated in the design of the study and the manuscript. Both authors read and approved the final manuscript.

## Additional files

File name: additional_file_1

File format: PDF

Title of data: Graphical illustration of the experimental approach

Description of data:

The additional data (see Additional file 1) provide a graphical overview of the experimental approach by explaining the position of the implanted electrodes within the PAG together with the ipsi- and contralateral alignments of the PLA electrodes. Additionally the removable connectors used on the animals are illustrated.

## Supplementary Material

Additional File 1 Graphical illustration of the experimental approach. Click here for file

## Figures and Tables

**Table 1:** Onset of KA-induced total suppression and recovery of PAG-elicited calls as shown in Figs. 2 and 3.

***Call_type (PAG)***	***KA_I/C (PLA)***	***T*_*supp *_ *[min]***	***T*_*rec *_ *[min]***	**Fig.**
EC	I	-	-	2A
CC	C	-	-	2A
EC	C	75	>280	2B
CC	I	-	-	2B
EC	I/C	24	>240	2C
CC	I/C	-	-	2C
EC	I	-	-	3A
EC	C	33	>140	3A
EC	I	-	-	3B
EC	C	8	110	3B

*Call_type *is either echolocation (EC) or communication (CC) call. KA application (*KA_I/C*) was either ipsi- (I) and/or contralateral (C) to the stimulation site in PAG. *T*_*supp*_, *T*_*rec *_are the times after the start of KA-application when the probability for eliciting calls was fully suppressed or fully recovered for a period of at least 10 min, respectively.
