# Supplementary material for: Echolocation calls and communication calls are controlled differentially in the brainstem of the bat Phyllostomus discolor
Source: BMC Biol. 2005 Aug 1;3:17. doi: 10.1186/1741-7007-3-17 (PMC1190161; doi:10.1186/1741-7007-3-17)
Supplement: Additional File 1 — Graphical illustration of the experimental approach. [file 1741-7007-3-17-S1.pdf]

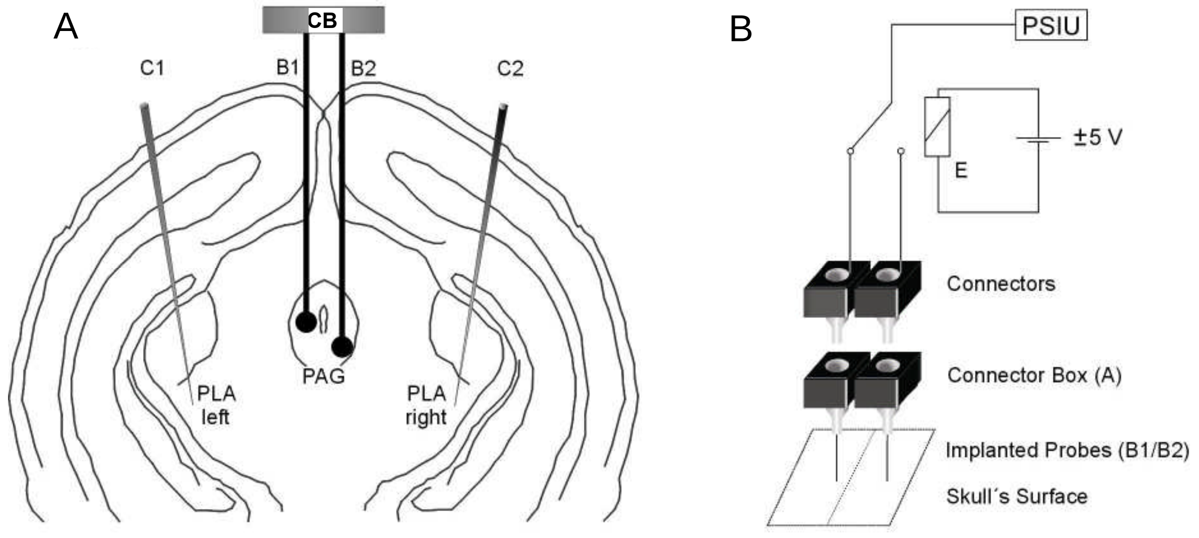

Additional_file_1: Graphical illustration of the experimental approach

Graph A illustrates the alignment of stimulation electrodes (*B1* and *B2*) within the PAG and the iontophoresis probes (*C1* and *C2)* within both PLA sites. *B1* and *B2* either both triggered echolocation calls or *B1* triggered a communication call while *B2* triggered an echolocation call, respectively. The type of the call elicited by the electrode depends on the position of the electrode tip within the PAG. Generally speaking when *B1* is active, *C1* is the ipsilateral probe and *C2* is the contralateral probe. During activation of *B2*, *C2* is the ipsilateral probe while *C1* is referred to as the contralateral probe. CB represents the connector box. Graph B illustrates the detailed setup of the connector box mounted on the skull of the animal. The remotely controlled relay was mounted between the isolation unit (PSIU) of the stimulator and the jack for the connector box, and facilitated an alternation of the desired call type without disturbing the animal.
